# Case Report: COVID-19 Pneumonia Following Left Pneumonectomy for Lung Cancer Complicated by Empyema and Bronchopleural Fistula

**DOI:** 10.3389/fsurg.2021.679757

**Published:** 2021-05-21

**Authors:** Alberto Testori, Veronica M. Giudici, Marco Alloisio, Ugo Cioffi

**Affiliations:** ^1^Department of General and Thoracic Surgery, IRCCS Humanitas Research Hospital, Milan, Italy; ^2^Department of General and Thoracic Surgery, Humanitas University, Milan, Italy; ^3^Department of Surgery, University of Milan, Milan, Italy

**Keywords:** COVID-19 pneumonia, pneumonectomy, thromboembolism, thromboprophylaxis, bronchopleural fistula, empyema

## Abstract

**Background:** Venous and arterial thromboembolism is commonly reported in critically ill COVID-19 patients, although there are still no definitive statistical data regarding its incidence.

**Case presentation:** we report a case of a patient who fell ill with Covid during hospitalization for a pneumonectomy complicated by empyema and bronchopleural fistula. The patient, despite being cured of COVID, died after 14 days for pulmonary thromboembolism.

**Conclusion:** Our case strengthens the suggestion of adequate thromboprophylaxis in all hospitalized COVID patients and of increasing prophylaxis in critically ill patients even in the absence of randomized studies

## Introduction

Patients with COVID-19 pneumonia exhibit a range of abnormal coagulation parameters resulting in increased mortality rate. Alterations of the hemostatic system include increased D-dimer and fibrin degradation products, changes in activated partial thromboplastin time (aPTT), and prothrombin time international normalized ratio (INR).

COVID-19 can predispose to venous and arterial thromboembolic disease as a result of excessive inflammation, hypoxia, immobilization and diffuse intravascular coagulation (1). Exact knowledge of the incidence of thrombotic complications in COVID-19 patients is important for decision making regarding the intensity of thromboprophylaxis, especially in ICU patients who are at higher risk for thrombosis.

Here, we report a case of a patient who fell ill with Covid during hospitalization for a pneumonectomy. The patient, despite being cured of COVID, later died of probable pulmonary thromboembolism.

## Case Presentation

**Figure 1:**
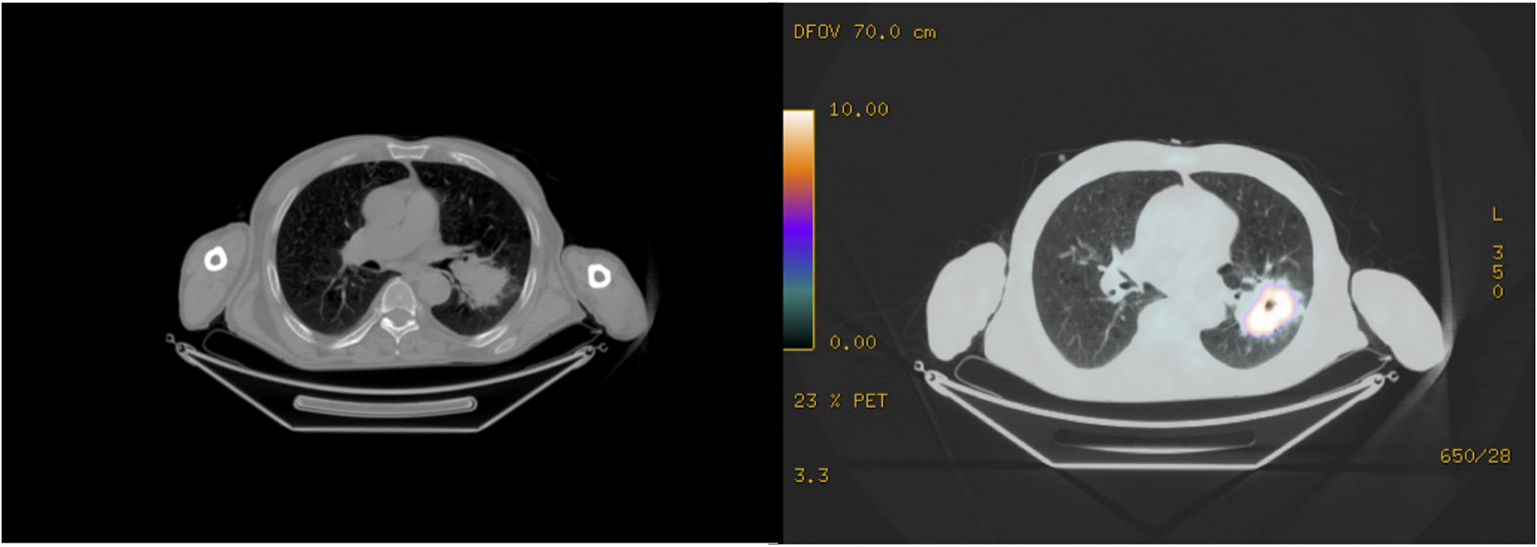
A PET-CT scan revealing the presence of a 7-cm left lung neoformation with no nodal involvement.

**Figure 2:**
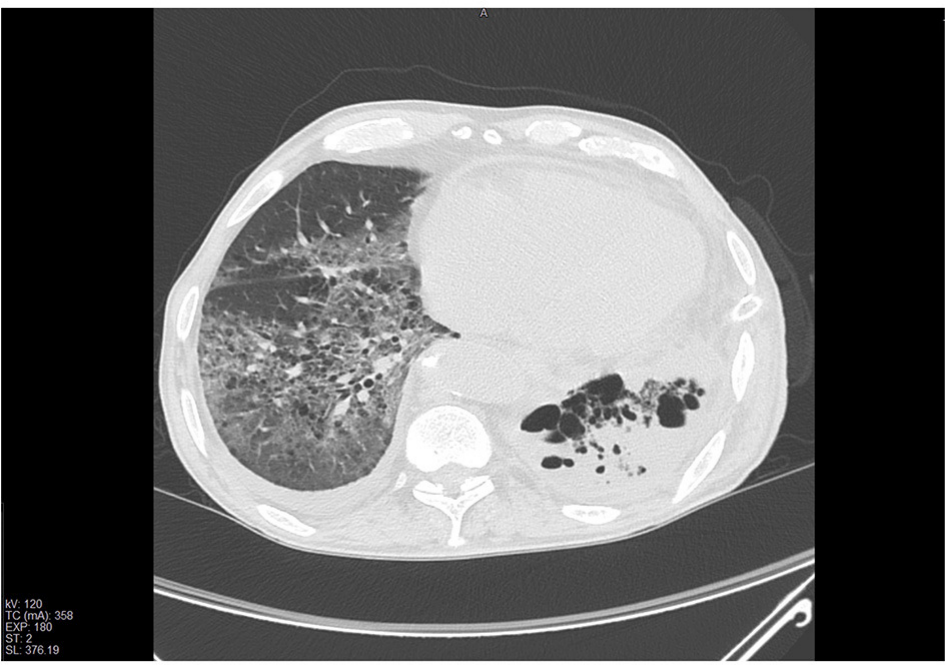
Chest CT-scan showing the presence of extensive pulmonary infarction in the residual parenchyma.

A 78-year-old man, who previously underwent to a radical cystectomy, was diagnosed of a left lung mass during a follow up with chest X-ray. A chest-CT, a PET-CT scan (Figure 1) and a CT guided fine needle aspiration biopsy revealed the presence of a 7-cm lung Adenocarcinoma, with no lymph nodal involvement and/or metastasis. So, the patient underwent to a lower left lobectomy surgery and a typical lingulectomy in anterolateral thoracotomy. Given the increase in inflammation indices and the progressive increase in opacity of the residual left lung parenchyma due to infarction (Figure 2), the patient was submitted to a left pneumonectomy. The hospital stay lasted 10 days and the patient was discharged in good clinical conditions. The final histopathological diagnosis was a 7,5-cm lung Adenocarcinoma; the bronchial surgical margin was negative and visceral pleural invasion was not observed. Of the 12 dissected lymph nodes, all were reported as negative and the pathological stage was reported as IIIA (T4N0M0).

**Figure 3:**
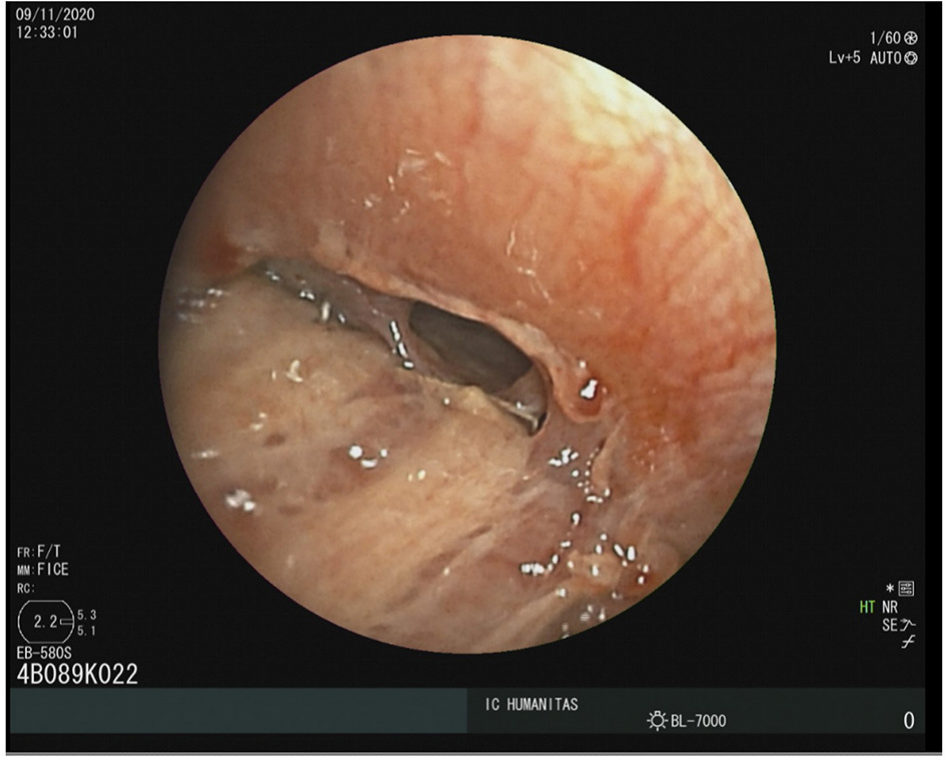
A flexible bronchoscopy showing a 4-mm bronchopleural fistula.

Four days later, he was readmitted to the ward due to fever and subcutaneous emphysema. A chest CT scan was performed, it showed the presence of a left abundant pleural effusion. A chest tube drainage was placed, an Enterococcus faecium was isolated from pleural fluid a therapy with linezolid 600 mg/twice a day intravenously was set. A 4-mm bronchopleural fistula was diagnosed with a flexible bronchoscopy (Figure 3) and the patient underwent to endoscopic lipofilling treatment (2). A nasopharyngeal COVID swab was performed and resulted positively, the patient was immediately transferred in COVID-ward.

**Figure 4:**
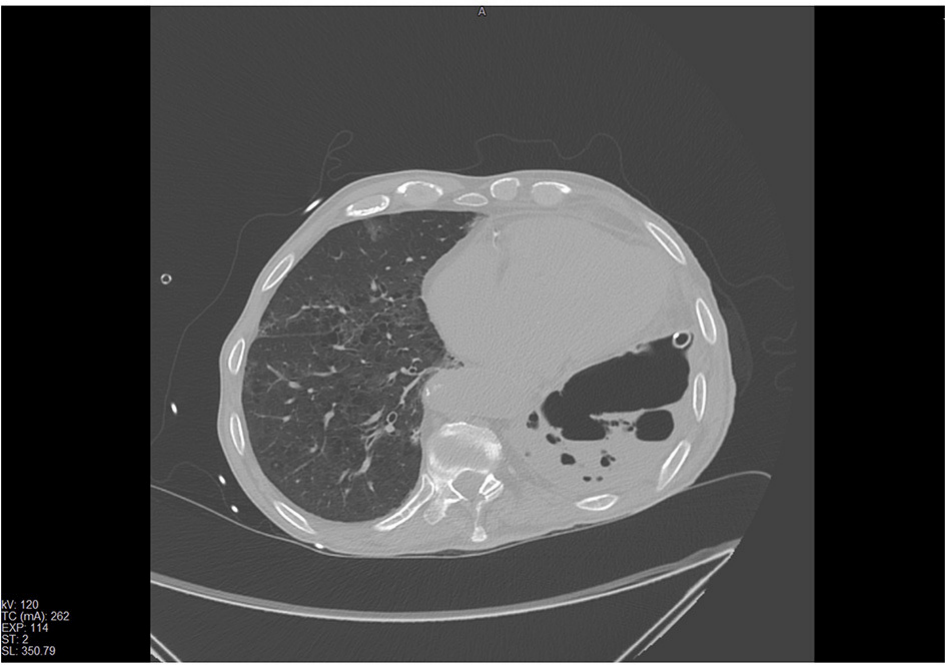
Chest CT scan revealing a non-homogeneous increase in density, diffuse GGO and consolidations at the right lung.

The patient developed dyspnoea, chest tightness and wheezing. Two days later, due to the worsening of clinical conditions he underwent to a chest CT-scan in which almost all of the right lung had a non-homogeneous increase in density, with diffuse ground glass opacity (GGO) and consolidations (Figure 4). With nasal oxygen, SpO2 was between 85 and 90%, so Venturi mask was introduced with 60% of FO2. His level of LDH was 321 and of D-Dimer 1,785 ng/ml. Remdesivir associated with steroids were introduced, antibiotic therapy was updated introducing meropenem 3 gr once a day intravenously.

**Figure 5:**
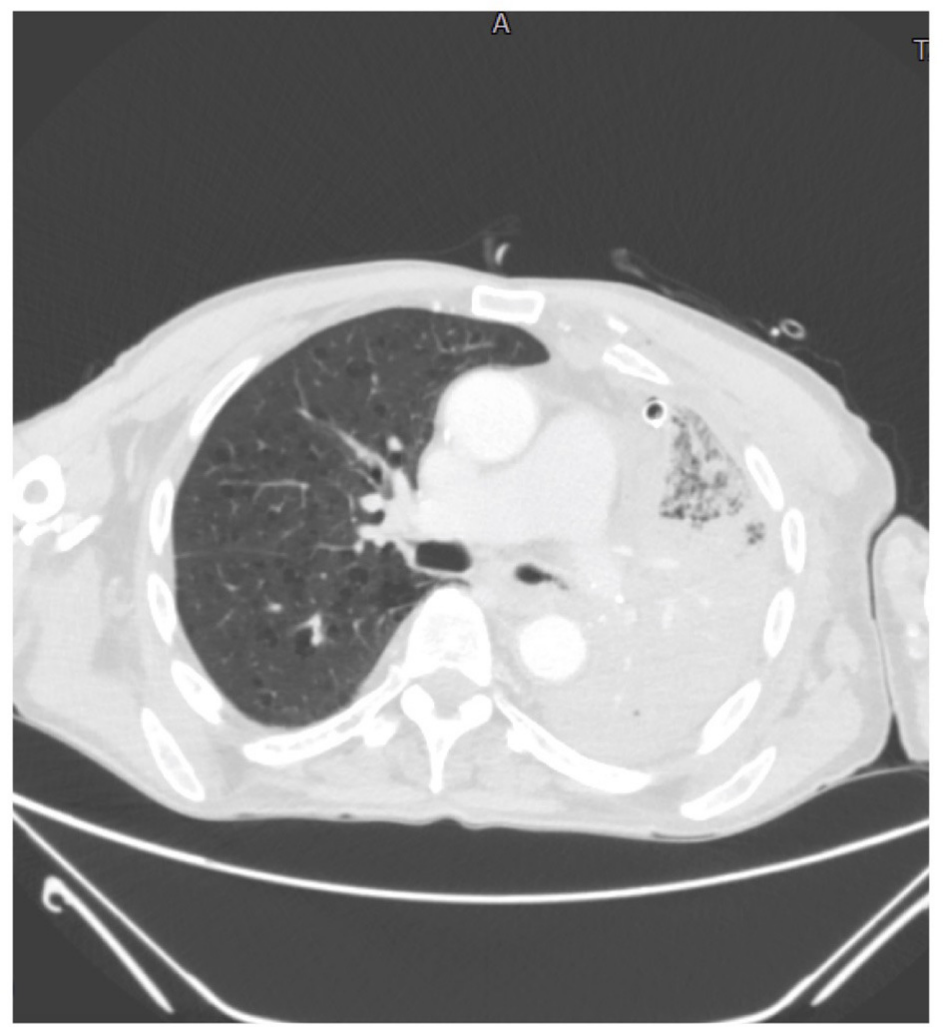
An almost complete recovery revealed by a new chest CT scan.

After a successful 14-day period of treatment with a constant improvement of respiratory conditions, a new nasopharyngeal swab was performed and resulted negatively. White blood cell and lymphocyte counts were normal, the C-reactive protein levels continued to regress, there was no important biochemical abnormality (LDH 208 and D-Dimer 645), with nasal oxygen 2l, SpO2 was between 97 and 100%. The patient underwent to a new chest CT-scan, with an almost complete recovery (Figure 5). The patient was transferred in a dedicated ward of ex-COVID, and he was scheduled for a new procedure of lipofilling in bronchoscopy.

Following an increase in inflammation parameters, a new sample of pleural fluid was taken from the thoracic drainage, with isolation of Pseudomonas aeruginosa and Candida albicans and a new antibiotic therapy was setting with levofloxacin 500 mg, once a day and piperacillin/tazobactam 4,5 g/3 times a day. Death occurred 14 days later due to cardio-circulatory arrest refractory to resuscitation maneuvers. The autopsy was not done because the relatives did not give consent and also because the echocardiogram performed during the resuscitation maneuvers showed that the patient suffered from massive intracardiac coagulation.

## Discussion

During COVID-19 pandemic, like previously described in China, we faced a reduction or even a suspension of elective surgery, due to the reduction of the availability of hospital beds, especially in intensive-care units (ICUs), and an increasing number of infections between health people (2). Is stated, also, that COVID-19 that develops in perioperative time is a risk factor for increased length of stay, morbidity and mortality, especially in thoracic surgery, were the lung, is manipulated to perform parenchymal resection (3, 4).

Various studies worldwide stated that hospitalized, ill coronavirus disease 2019 (COVID-19) patients are frequently developing laboratory abnormalities compatible with a state of hypercoagulability and clinically a high prevalence of thromboembolic events (5). Ranucci et al. (1) recently reported a coagulation analyses including d-dimers, fibrinogen levels, in the COVID-19 patients, and reported the procoagulant profile on ICU admission with median d-dimer levels 10 times the upper limit of normal (5.5 mg/L).

Although the number of postmortem pathologic reports are limited, vascular wall thickening, stenosis of the vascular lumen, and microthrombus formation accompanying the findings of ARDS have been reported by Luo et al. (6).

Hypercoagulation has also been described in the systemic circulation, have been described thrombosis and major thromboembolic sequelae including Pulmonary Embolism in 20–30% of ICU patients due to hypercoagulability with hyperfibrinogenemia (7, 8).

The effect of heparin, mainly LMW heparin, is reported by Tang et al. (9) showing reduced mortality in cases with coagulopathy treated with heparin compared to patients who had coagulopathy not treated with heparin (40.0 vs. 64, 2%, respectively; *p* = 0.029). Heparin exhibits anti-inflammatory effects by neutralizing DAMPs to protect endothelial cells and reduce pulmonary edema and vascular loss.

In the clinical case we treated, the patient was subjected to a prophylactic low-molecular-weight heparin therapy, already before contracting COVID.

The heparin dosage for patients undergoing oncological thoracic surgery is established according to the Caprini score. This score is based on several factors including age, weight, a history of thromboembolism, prolonged immobilization. According to the Caprini Score, our oncological patients, who are undergoing surgery, receive low molecular weight subcutaneous heparin at a dosage of 0.3 ml, 2,850 Ul the night before surgery until 2 days after. From the third postoperative day, the dosage is increased to 0.4 ml, 3,800 Ul or 0.6 ml, 5,700 Ul for up to 25 days after surgery or until complete mobilization.

However, the question at this point is: is this dosage enough for oncological patients who contract COVID?

For oncological patients, who already have an increased thrombotic risk, it would be better to consider an increase in the heparin dosage (10). Therefore, not only for prophylactic but also for anticoagulant purposes.

## Data Availability Statement

The original contributions presented in the study are included in the article/supplementary material, further inquiries can be directed to the corresponding author/s.

## Ethics Statement

Ethical approval was not provided for this study on human participants because case report. The patients/participants provided their written informed consent to participate in this study. Written informed consent was obtained from the patient for publication of this case report and any accompanying images.

## Author Contributions

All authors listed have made a substantial, direct and intellectual contribution to the work, and approved it for publication.

## Conflict of Interest

The authors declare that the research was conducted in the absence of any commercial or financial relationships that could be construed as a potential conflict of interest.
